# Heteroatom-Doped Metal-Free Carbon Nanomaterials as Potential Electrocatalysts

**DOI:** 10.3390/molecules27030670

**Published:** 2022-01-20

**Authors:** Jayeeta Chattopadhyay, Tara Sankar Pathak, Daewon Pak

**Affiliations:** 1 Chemistry Department, Amity University Jharkhand, Ranchi 834002, India; 2 Department of Science and Humanities, Surendra Institute of Engineering and Management, Siliguri, Darjeeling 734009, India; drtspathak1971@gmail.com; 3 Department of Environmental Engineering, Seoul National University of Science and Technology, Gongneung-ro, Nowon-gu, Seoul 01811, Korea

**Keywords:** carbon nanomaterials, nitrogen doping, sulphur doping, co-doping, electrocatalysts

## Abstract

In recent years, heteroatom-incorporated specially structured metal-free carbon nanomaterials have drawn huge attention among researchers. In comparison to the undoped carbon nanomaterials, heteroatoms such as nitrogen-, sulphur-, boron-, phosphorous-, etc., incorporated nanomaterials have become well-accepted as potential electrocatalysts in water splitting, supercapacitors and dye-sensitized solar cells. This review puts special emphasis on the most popular synthetic strategies of heteroatom-doped and co-doped metal-free carbon nanomaterials, viz., chemical vapor deposition, pyrolysis, solvothermal process, etc., utilized in last two decades. These specially structured nanomaterials’ extensive applications as potential electrocatalysts are taken into consideration in this article. Their comparative enhancement of electrocatalytic performance with incorporation of heteroatoms has also been discussed.

## 1. Introduction

Energy demand and energy production are continuously establishing a disproportional relation to each other, which leads to a crucial environmental crisis globally. The over consumption of fossil fuels and excessive CO_2_ emission results in global warming, and consequently, the scientific community is being faced with the most challenging situation in mitigating this serious environmental crisis [1,2,3]. Moving towards this aim, fuel cells, metal–air batteries, water electrolysers, rechargeable batteries and electrochemical capacitors constitute various technologies in respect to energy production/storage [4]. In recent years, these applications are mainly covered with various research works on oxygen reduction reaction (ORR), oxygen evolution reaction (OER), hydrogen evolution reaction (HER) and electro-reduction reaction of carbon dioxide (CO_2_RR) [5,6,7,8]. However, these electrochemical processes are largely restricted due to their high activation energy barriers, especially in oxygen reduction reaction (ORR) kinetics performed at the cathode [9,10]. The much slower cathodic ORR limits the overall output performance of these useful technologies [11]. Usually, electrocatalysts play a pivotal role in reducing the activation energy barriers of ORR process. In last two decades, platinum (Pt)-based materials are continuously considered the most potential electrocatalysts in this regard, leading to relatively higher current density and lower overpotential value [12,13,14,15]. However, large-scale application of these materials was restricted due to their cost ineffectiveness, easy dissolution of Pt, instability due to CO deactivation and fuel crossover effect. For these reasons, today, researchers are more inclined to develop electrocatalysts with the following priorities: minimization of the Pt metal loading; alloying of Pt with other transition metals to improve catalytic performance (Pt–Co, Pt–Ni, Pt–Fe, Pt–Ru, Pt–Pd, Pt–Rh, Pt-TiO_2_ and Pt–Sn catalysts) [16,17,18]; advanced nonprecious metals and metal oxides [19,20,21,22,23]; metal-incorporated carbon materials [24,25,26,27]; and even metal-free catalysts [28,29,30] with remarkable electrocatalytic performance, enhanced durability and greater electrochemical stability with satisfying cost-effectiveness. The nonprecious metal-based catalysts always show lower catalytic activity in comparison to Pt/C, and they also show poor durability due to metal leaching during application. In contrast, the metal-free carbon-based materials perform excellent catalytic activity during ORR [31,32,33,34,35]. Moreover, they are extraordinarily chemically stabile, cost-effective, and environmentally friendly. These remarkable properties make various carbon nanostructures, viz., graphene, carbon nanotubes (CNTs) and carbon nanohorns (CNHs), hugely popular in many potential applications. The family of carbon allotropes is mainly constituted of a huge sp^2^ lattice, which is an extended π conjugation system, resulting in greater thermal and electrical conductivity. Moreover, these specially structured materials act as a substrate in various covalent and noncovalent modifications on their lattice structure, which results in the improvement of their inherent characteristics and adoption of new ones [36,37,38,39].

**Scheme 1:**
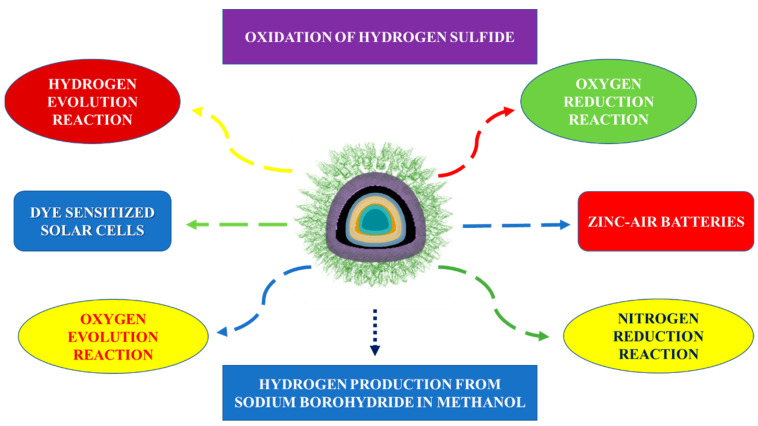
Schematic representation of hetero-atom doped metal-free electrocatalysts in different potential application fields.

**Figure 1:**
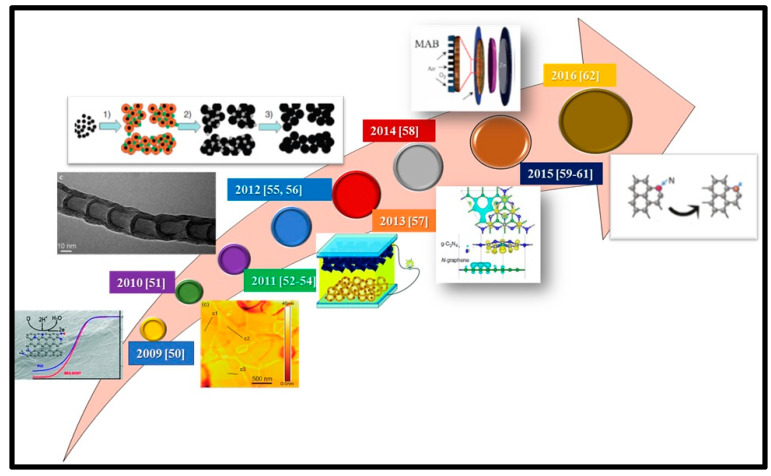

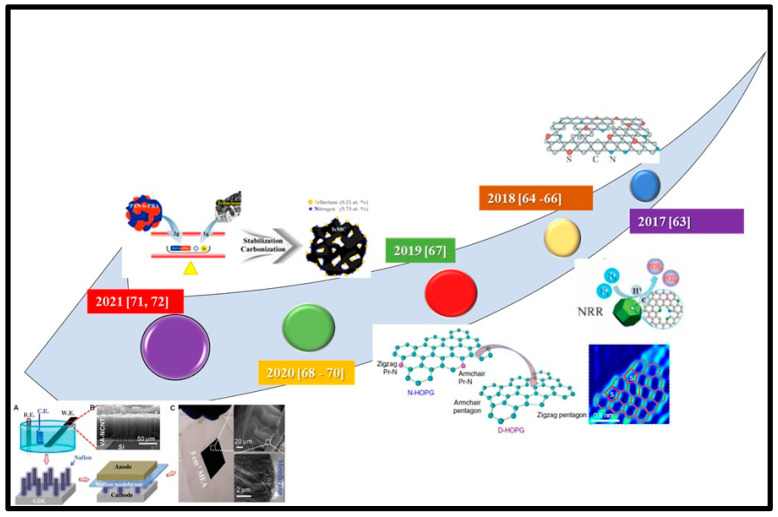
(**a**) [50,51,52,53,54,55,56,57,58,59,60,61,62] and (**b**) [63,64,65,66,67,68,69,70,71,72]: Developments of heteroatoms incorporated metal-free carbon nanomaterials in last two decades.

The carbon nanomaterials can be functionalized with the alteration of their surface, interfacial structure and electronic properties, extending their applicability to higher levels. One of the most cutting-edge functionalization technologies is chemical doping, when carbon lattice is enriched with heteroatoms. They can be incorporated in the adsorptive form (in the case of metal atoms), which leaves the sp^2^ lattice intact. However, heteroatoms of similar radius to C, e.g., N, O, P, S, B, etc., are used to bind different configurations, where substantial effect can be seen through sp^3^ defects. Incorporation of heteroatoms, which are more electronegative than C, can polarize the sp^2^ network accordingly, therefore resulting into novel electrochemical properties. In last two decades, introduction of heteroatoms into the graphitic framework structures garnered great attention from researchers. Boron, nitrogen, phosphorus, sulphur, fluorine, chlorine, bromine, iodine, selenium, antimony and tellurium have widely been used as heteroatoms [40,41,42,43,44,45,46,47,48,49]. Several reviews have been published in last few years on heteroatom-doped nanomaterials applied suitably in ORR; however, very few scientists have covered their overall applicability in other electrochemical reactions. This review covers heteroatom doping on metal-free carbon nanomaterials as a potential electrocatalyst, discussing the future challenges and perspectives in this rapidly evolving field. In this work, special attention is also paid to their synthetic strategies and how their structural orientation could affect their applications as electrocatalysts. Scheme 1 represented the various types of elecctrocatalytic applications of hetero-atom doped metal-free nano-materials. This article is not restricted to discussing their applications in oxygen reduction reaction only; we also investigate their applications in all types of electrochemical processes. This review work also provides a brief overview of the gradual research progress of these materials in last two decades and the challenges confronted in potential applications of them as metal-free electrocatalysts. Figure 1a,b and Table 1 described the developments of heteroatoms incorporated metal-free carbon nanomaterials and their potential applications in different electrocatalytic activities in last two decades.

## 2. Nitrogen-Doped Metal-Free Carbon Nanostructured Electrocatalysts

### 2.1. Nitrogen-Doped Carbon Nanotube Electrocatalysts

**Figure 2:**
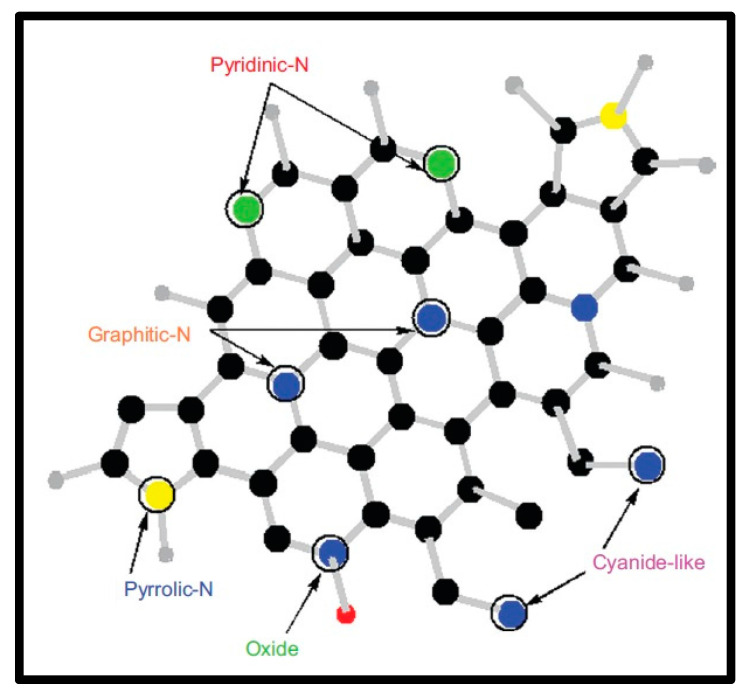
Nitrogen doping in the carbon nanomaterial structures (Reproduced with permission from S. Majeed et al. [89]).

The functionalized nanotubes garnered significant attention in the field of the reinforced and conductive plastics, sensing materials and photovoltaic materials as scanning probe microscopy tips and many more applications. There have been two broad methods to synthesize substituted N-doped CNTs: (a) in situ process for insertion of nitrogen atom into the CNTs during the reaction, only [74,75,76,77,78]; and (b) postfunctionalization of CNTs with nitrogen by using various precursors and compounds like organic moieties. However, the postfunctionalization method has not been well investigated until now [79,80,81]. These nanomaterials have also been synthesized using other potential synthetic strategies, viz., arc discharge, laser ablation and plasma etching [82,83,84,85,86,87]. However, these methodologies required higher temperature conditions and a limited type of nitrogen or carbon precursors. Moreover, rapid evaporation of precursors and application of nitrogen or ammonia atmosphere has been required. In the chemical vapor deposition (CVD) method, the process could completed at a lower temperature range with and without the presence of an organometallic catalyst and by using a wide range of carbon or nitrogen precursors. This method could produce 20–25 g of N-carbon nanotubes per gram of catalyst, and nitrogen atoms are embedded into the hexagonal carbon network at various ratios with 10 atoms [81]. In the literature, nitrogen incorporation has been reported with nitrogen contents of <1 atom% to 20 atom% [75,88,89]. Highly oriented nanotubes with regular diameter and bond-length were termed in literature as “carpet-like” structures [90]. In this work, nitrogen was incorporated into the already synthesized CNT structure; however, this synthesis method was depicted as highly complex and tedious with multistep techniques. The first step is initiated with a chemical oxidation process of tips or structural defects of CNTs, followed by coupling with other molecules through carboxylic, carbonyl and/or hydroxyl groups. The covalent functionalization via bond formation to the π-conjugated structure of CNT leads to the rehybridization of sp^2^ bond to sp^3^. In this type of structure, nitrogen is attached to carbon following two different manners: (a) pyridine-type nitrogen, in which each nitrogen atom is bonded to two different carbon atoms, leading to the formation of cavities within the side wall of the tube, and (b) substitution N, in which a nitrogen atom bonds with three C atoms, as presented in Figure 2. Nitrogen contains an additional electron in its structure, in comparison to the carbon network; therefore, a nitrogen-incorporated CNT structure usually exhibits metallic properties [90,91,92]. The nitrogen group can also enhance the reactivity on the graphene in comparison to the pure CNT structures, which results in the potential applications of these materials in fast-responsive sensing technology; as effective field-emissions sources; and as polystyrene, epoxy composites, protein and nanoparticle immobilizers [78,93,94,95,96]. The most popular covalent functionality, with the application of plasma etching or by HNO_3_/H_2_SO_4_ treatment, includes carbonyl or carboxyl groups [97].

The plasma etching technique is essentially applicable during functionalization processes in a nitrogen atmosphere. In the next step, carboxyl groups are acylated with thionyl chloride to establish a basis for different amine compounds [98] or to combine with DNA and proteins [99,100]. The noncovalent functionalization is mostly conducted by adsorption or through the wrapping of the CNTs in polymer polynuclear aromatic compounds, surfactants or biomolecules by Vander Waals forces and π–π interactive forces. Other synthetic approaches of CNTs include the arc evaporation method of graphite [82,101]. The noncovalent methods are more favourable over covalent, as the chemical functionalization can be performed on the CNTs without affecting their structures and electronic networks on the nanotubular structures.

#### 2.1.1. Chemical Vapour Deposition (CVD) Method

Chemical vapour deposition (CVD) is a technique to synthesize carbon nanotubes in bulk amounts, which involves the pyrolysis of different organic molecules, viz., CH_4_, C_6_H_6_, C_2_H_2_, etc., in inert atmosphere over Ni, Co, Fe, etc., catalysts [102,103]. Due to the simplicity and cost-effectiveness of CVD, researchers prefer to follow this methodology during the functionalization process.

**Figure 3:**
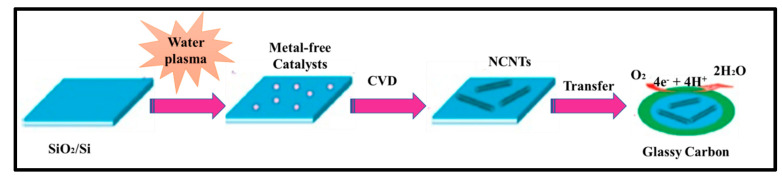
N-doped metal-free CNTs as ORR electrocatalysts (Reproduced with permission from Chen et al. [86]).

**Figure 4:**
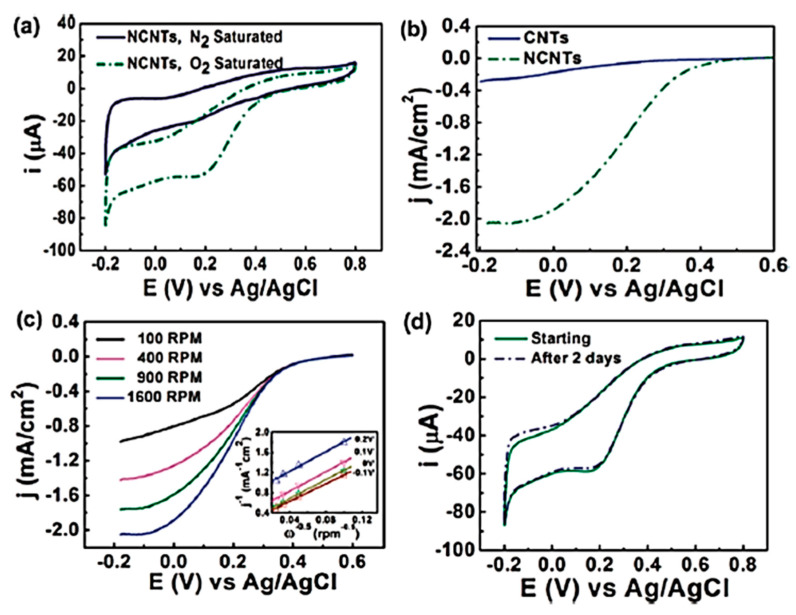
Electrochemical studies in ORR under acidic medium with N-doped metal – free CNTs (Reproduced with permission from Yu et al. [86]).

**Figure 5:**
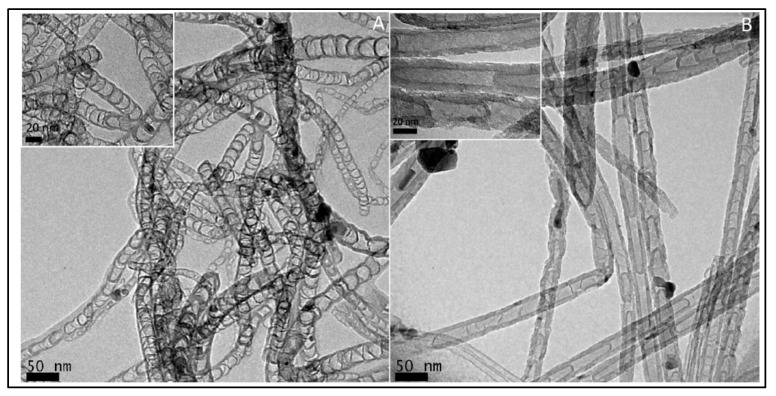
TEM images of bamboo-structured NCNTs (**A**) ethylenediamine–NCNTs (**B**) Pyridine–NCNTs (Reproduced with permission from Chen et al. [50]).

In 1997, Dai et al. introduced H_2_O plasma etching technology to generate surface patterns of polar groups with oxygen [104]. This methodology was further followed by Yu et al. [86] to develop SiO_2_ nanoparticles as the metal-free catalysts, in which a SiO_2_/Si wafer with a 30 nm-thick SiO_2_ coating was employed with H_2_O plasma etching at 30 W, 250 kHz and 0.62 Torr for 20 min. This plasma-etched substrate was placed into a tube furnace for the synthesis of CNTs by using the CVD method. Figure 3 represents the schematic diagram to represent the growth of CNTs. These materials acted as potential electrocatalysts in an oxygen reduction reaction analysed in 0.5 M H_2_SO_4_ solution saturated with N_2_ or O_2_. Figure 4 shows the various electrochemical studies conducted in this work. All the electrocatalytic studies have shown excellent results and long-term stability in an acidic medium in comparison to undoped CNTs. The authors also claimed that, due to the highly generic nature of the plasma etching technique, this synthetic strategy can be well accepted in various fields, from energy applications to electronic and biomedical systems [86]. Kim et al. mentioned a similar synthesis process in an Ar atmosphere at 800 °C for 1 h duration in which ferrocene, pyridine or ethylenediamine used as a catalyst carbon and nitrogen precursor, respectively [50]. TEM images of bamboo-structured N-doped CNTs (NCNTs) are presented in Figure 5. These products were used as excellent electrocatalysts in ORR of fuel cell applications. The same research group reported synthesis of nitrogen-doped CNTs by following a single step the CVD method in which either ferrocene or iron (II) phthalocyanine was used as the catalyst and pyridine as the carbon and nitrogen precursor, respectively. These materials have also used successfully as ORR electrocatalysts [105].

**Figure 6:**
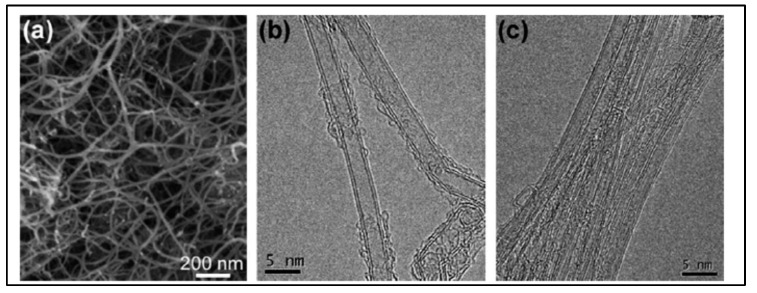
(**a**) SEM image shows the nanotube bundles with a diameter of 10–20 nm (**b**) HRTEM image reveals that the bundles are consisted mainly of double walled CNTs DWNTs. (**c**) A magnified image showing the outer diameter in the range of 1.5–2 nm (Reproduced with permission from Kim et al. [109]).

In 2011, Feng et al. [52] reported N-doped CNTs to be effective electrocatalysts in microbial fuel cells (MFCs), boasting cost-effectiveness and long durability. Moreover, these materials were depicted as more effective cathodic catalysts than the commonly used platinum catalyst with a maximum power density value of 1600 ± 50 mW·m^−2^. These N-doped CNTs have shown a lower drop in the percentage of power density than that of Pt/C over 25 cycles. Another research group reported the CVD synthesis floating catalyst method of nitrogen-doped carbon nanotubes using ferrocene/aniline together with toluene as an added carbon source [106]. Yang et al. synthesized aligned nitrogen-doped CNT bundles over 700–800 °C by taking ammonium-exchanged zeolite-β as the substrate material, ferric nitrate as the catalyst and acetonitrile as the carbon precursor [107]. In the same year, He et al. reported controllable synthesis of aligned CNx with a large surface area by pyrolyzing CH_3_CN/Fe(C_5_H_5_)_2_ on SiO_2_ and Si substrates over the temperature range of 750–900 °C. The specific diameters of CNTs diminished on Si substrates in comparison to a well-documented rise with temperature on silica, as the growth process followed different mechanisms of formation of catalyst particles [108]. Kim et al. developed N-doped, double-walled CNTs using chemical vapor deposition in which a CH_4_/NH_3_/Ar mixture flowed with the rate of 50/10/500 sccm on MgO-supported catalyst powders at the temperature of 850 °C for 10–30 min. of duration [109]. The synthesized CNTs are formed with a diameter of 10–20 nm. The SEM and HRTEM images of N-doped double walled-CNTs presented in Figure 6. 

**Figure 7:**
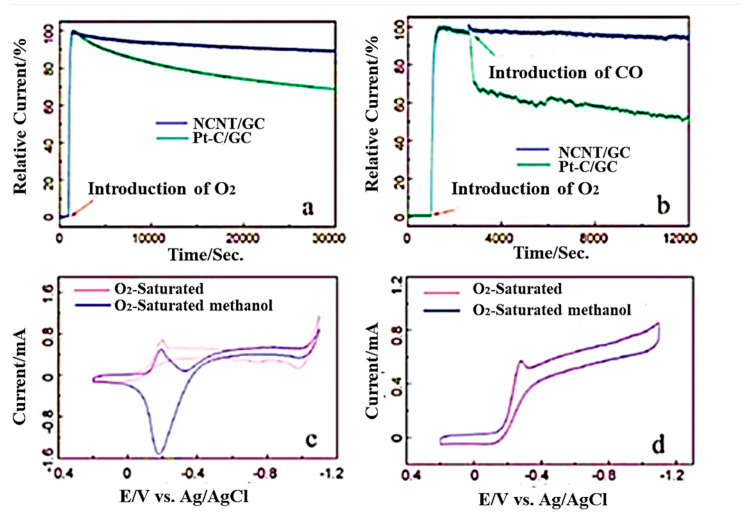
(**a**) Chronoamperometric studies with NCNT/GC and Pt–C/GC electrodes in oxygen saturated 0.1M KOH. (**b**) Responses after the introduction of 10% CO. (**c**) Cyclic voltammograms of Pt/GC and (**d**) cyclic voltammograms of NCNT/GC electrodes oxygen saturated 0.1 M KOH, with and without 3 M methanol solution (Reproduced with permission from Wang et al. [116]).

In recent years, Li et al. reported a one-step CVD method to synthesize three-dimensional nitrogen-doped CNT/graphene hybrid material on nickel foam [110]. In this study, nickel foam and melamine were mixed with the mass ratio of 1:5 kept in a horizontal quartz tube reactor and heated to within the temperature range of 600–800 °C in a hydrogen atmosphere for around 20 min at a flow rate of 70 sccm. In 2020, another research group mentioned a two-step synthetic strategy to develop nitrogen-doped carbon nanotubes derived from g-C_3_N_4_ [111]. In this case, exfoliated graphitic carbon nitride was functionalized with nickel oxides and placed in a ceramic boat to keep in the tubular furnace at 900 °C in a nitrogen atmosphere. Hydrogen was introduced for 3 h in the first step and ethylene for 10 min for the reduction process. The synthesis of N-doped MWCNTs with straight structure was reported by Xu et al. by using phthalocyanine derivatives [112] and the mixture of ethylene/hydrogen and ammonia at around 680 °C in a presence of alumina-supported iron catalysts in a CVD furnace [113]. The amount of nitrogen incorporated into CNT can be controlled by using different amounts of nitrogen precursors [80,114]. The rate in which nanotubes grow during synthesis can be enhanced with the increase in its precursor significantly, resulting into the increase in intensity ratio of the D to G bands in Raman spectra. The inner structure of N-doped CNTs constitutes regular morphological transformation from the straight and smoother walls (0 atom% N) to 1.5 atom% N-containing, bamboo-structured CNTs; further, it changed to corrugated structures with 3.1 atom% and higher nitrogen [115]. It has been analysed by Wang et al. that, during the synthesis of N-doped CNTs, when melamine is used as the C/N initiator, it can incorporate 20 atom% nitrogen. In this type of synthesis method, the N atom present in the reaction medium self-assembled with gaseous carbon without any assistance from metal [116]. These N-doped CNTs were utilized successfully as ORR electrocatalysts in methanol fuel cells measured in alkaline media. Figure 7 shown the various electrochemical studies of NCNT/GC. 

The incorporation of nitrogen atoms usually shows very strong ability to promote the self-assembled CNTs. Nitrogen could create highly active sites in carbon networks, which results in remarkable electrocatalytic performance comparable to traditional Pt-based materials as electrocatalysts. Their high activity with excellent stability and selectivity always made N-doped CNTs better electrocatalysts in this purpose. These materials were also strongly resistant to CO poisoning, had a robust structure and were economically favourable. Due to doping in CNT structures, a basic shape could be transformed from a hollow cylinder to a bamboo-shaped structure. The resultant doped materials contained plenty of compartments, the lengths of which gradually decreased with variation in N concentration [117].

#### 2.1.2. Chemical and Electrochemical Modification Method

The chemical modification methods to synthesize nitrogen-doped CNTs included two different approaches, viz., covalent and noncovalent. During the covalent modification, oxygen-containing functional groups, viz., carboxyl and hydroxyl, were formed and generated on the surface. Among the functional groups, carboxylic acid groups were chosen as the best options, as they could easily proceed a variety of reactions in the modification process and could be easily developed using different oxidizing treatments, e.g., ozonolysis, sonication in nitric and sulfuric acid, refluxing in nitric acid, etc. In the next step, carboxyl-functionalized CNTs were grafted with the functional moieties by using the terminal oxidation process following various mechanisms from the defect site, from chemistry oxidation reactions and esterification/amidation processes to the already oxidized CNTs [118,119], mechanochemical modification [120,121], ionic liquids, cycloaddition reactions [122,123], electrochemical modification reactions, diazotization [124] and radical additions [125].

The efficient and successful doping and tailoring technologies in CNTs involved the controlling of redox properties of the dopant. Nitrogen-doped CNTs have excellent electrocatalytic activity compared to Pt electrodes, which could be acclaimed by the formation of additional active sites on the surface of the materials and has led to better dispersion of the Pt particles over the N–CNT and performed better in methanol oxidation [126]. From the results, it was analysed that doped CNTs as electrode materials always enhanced the output power of the thermoelectrochemical cells. Doping enhanced the electrochemical active surface area (ESCA) values in the CNT electrodes in proportional way. Wei et al. reported doped CNTs mixed with glutaraldehyde functionalized chitosan (GCS), which depicted an improved biocompatibility and higher conductivity in enzyme immobilization process, due to the enhanced kinetics from the N–CNTs [127]. The electrochemical modification process was carried out through two types of coupling reactions, working under oxidative or reductive conditions. In 2002, Kooi et al. worked on anodic coupling reaction to the SWCNTs by using two different aromatic amines, viz., 4-aminobenzylamine and 4-aminobenzoic acid [128]. The noncovalent functionalization process could be carried out through the porphyrin assembled on the N-doped MWCNTs via the Fe-N coordination. Tu et al. reported this noncovalent modification by porphyrin, which led the MWNTs insoluble in water, however, performed well as catalysts and biosensors [129].

### 2.2. Nitrogen-Doped Carbon Hollow Spheres

The carbon spheres usually referred to the spherical shaped carbon in semicrystalline or crystalline form, and constituted solid, hollow or core-shell morphological structures. Researchers have paid huge attention to nitrogen-doped hollow spherical structures in recent years due to their lower density, greater surface area values, better electrical conductivity, and excellent structural stability. In 2012, Zhu et al. developed a hierarchical porous hollow carbon nanospheres as an oxygen reduction electrocatalyst for zinc–air batteries, which contained active pyridinic-N and graphitic-N by using polystyrene spheres and aniline as the corresponding template and precursor [130]. Gu et al. reported N-doped porous carbon spheres with excellent porosity characteristics, which was used as a potential electrocatalyst in ORR. The unique spherical structures with remarkable stability and recyclability made these materials the most promising ORR electrocatalysts [131]. Hydrothermal carbonization method was adopted to make these materials by using biomass glucose, followed by treatment in ammonia and by subsequent activation treatment. Another research group reported the development of N-doped carbon nanodots @ nanospheres, which were applied as efficient electrocatalyst in ORR, in which high electrocatalytic activity was shown with an onset potential of −0.08 V, and they showed greater durability and greater resistance to the methanol cross-over effect; these results were comparable to commercially available Pt/C electrocatalyst. These N-doped carbon nanodots of sizes 2–6 nm were successfully formed by using the hydrothermal method from natural biomass (e.g., fresh grass) at 180 °C for 10 h duration. Furthermore, these carbon nanodots were subsequently immobilized onto functionalized microporous carbon nanospheres (MCNSs) with an average diameter of ∼100 nm and a surface area of 241 m^2^ g^−1^ via a simple hydrothermal process to self-assemble a carbon-based nanocomposite (N-CNDs@MCNSs) in the presence of oxygen (O)-containing surface functional groups [132]. Today, a significant number of research works were carried out on nitrogen encapsulation on metal/metal oxides/carbon nanosphere materials potentially applied as electrodes or electrocatalysts [133,134,135,136,137,138,139,140]. In the current review, those works were not considered, as they are not metal-free nanostructured materials.

### 2.3. Nitrogen-Doped Graphene Electrocatalysts

**Figure 8:**
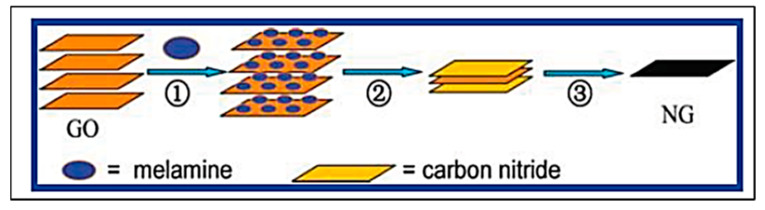
Schematic diagram of nitrogen doping method with melamine in the GO layer (Reproduced with permission from Sheng et al. [154]).

**Figure 9:**
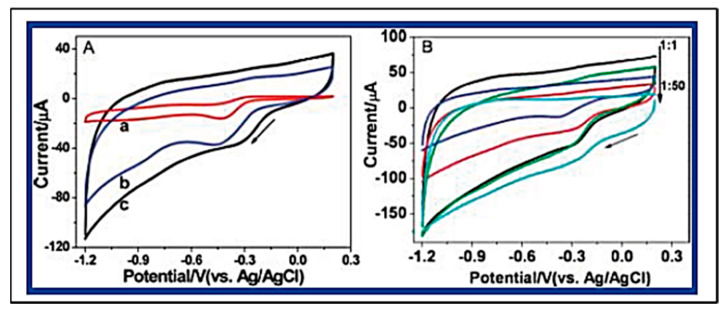
(**A**) Typical cyclic voltammograms (CVs) for ORR obtained at a bare GCE (a), graphene/GCE (b), and NG5/GCE (N% = 7.1%) (c) in O_2_ saturated 0.1 M KOH aqueous solution. (**B**) CVs for ORR at NGs, synthesized with different mass ratio of GO and melamine (1:1, 1:2, 1:5, 1:10, 1:50) at 800 °C, modified GCE in O_2_ saturated 0.1 M KOH aqueous solution. (Reproduced with permission from Sheng et al. [154]).

Graphene has been two-dimensionally structured with sp^2^ hybridized carbon with interesting physical and chemical characteristics. To achieve the desired performance in electrochemical and biochemical applications, nitrogen-enriched graphene materials were synthesized using a wide range of methodologies [141,142,143,144,145,146,147,148,149,150,151]. In 2011, Zhang et al. developed N-doped graphene by thermally annealing graphene oxide in the presence ammonia [152]. Another research group reported a facile and catalyst-free method to develop large-scale synthesis of nitrogen-doped graphene with 10.1 wt% nitrogen content by using the economically favourable industrial material melamine as the nitrogen source [153]. Sheng et al. synthesized nitrogen-doped graphene using the solvothermal method with the reaction between tetrachloromethane with lithium nitride under mild conditions [154]. Figure 8 and Figure 9 present the schematic diagram to synthesize these materials and their potential electrocatalytic applications in ORR under alkaline media, respectively. Another simple way to produce N-doped graphene nanosheets following the solvothermal route is via the reaction between graphene oxide and urea with a nitrogen content of 10.13 atom% [155]. Temperature played a pivotal role in the solvothermal process during the doping of nitrogen in the graphene network [152,153]. Another research group developed pyrrolic and pyridinic type nitrogen incorporation in a graphene structure at 300 and 500 °C, respectively, with the annealing treatment of graphene oxide in the presence of glycine and AgNO_3_ [156]. This particular methodology produced N-doped graphene with 13.5 atom% of nitrogen into the materials. The CVD method was also adopted by using methane and ammonia; these materials were utilized as metal-free electrocatalysts in ORR applied in fuel cells [157]. Many research groups have also applied the arc discharge method in H_2_ and He atmosphere and under pyridine vapour to produce the nitrogen-doped graphene structure [158,159].

**Figure 10:**
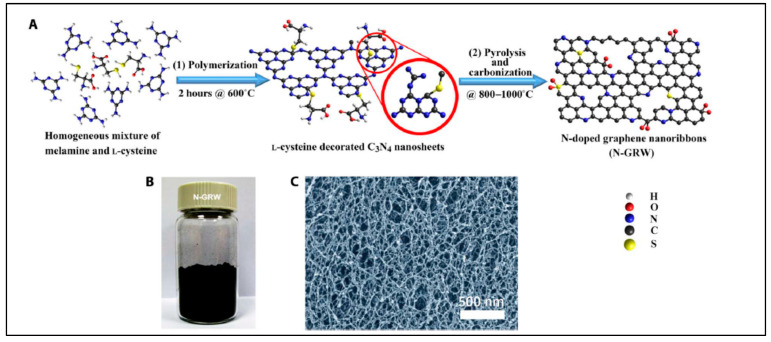
Synthesis of N-doped graphene nanoribbon networks (GRW). (**A**) Synthesis steps: (1) polymerization at 600 °C for 2 h, and (2) pyrolysis and carbonization at 800° to 1000 °C. (**B**) Digital photograph of the as-synthesized N-GRW. (**C**) Scanning electron microscopy (SEM) image of the as-synthesized N-GRW (Reproduced with permission from Yang et al. [160]).

Yang et al. reported synthesis of N-doped graphene, the result of which was successfully demonstrated as highly efficient metal-free bifunctional electrocatalysts in the oxygen reduction and evolution reaction [160]. In this report, e- donating quaternary nitrogen sites were responsible for ORR; in contrast, e- withdrawing pyridinic nitrogen acted as active sites in OER, resulting into greater transports of electrons and electrolytes [160]. The schematic diagram to synthesize N-doped graphene nanoribbon networks shown in Figure 10 [160]. 

Liu et al. synthesized pyrrolic–nitrogen doped graphene, which was successfully adapted as carbon-free electrocatalysts in electrocatalytic reduction of carbon dioxide to formic acid in their comparative study with the computational method [161]. Earlier, Ju et al. developed nitrogen-doped graphene nanoplatelets as potential metal-free, counter-electrode materials used in organic dye-sensitized solar cells [162]. Rahsepar et al. followed a hybrid hydrothermal-microwave process to synthesize N-doped graphene, which exhibited remarkable electrocatalytic activity in ORR [163]. The number of catalytic sites was enhanced due to the incorporation of N-atom into graphene. Maouche et al. developed nitrogen-doped graphene with porous structures, which was successfully employed as an ORR electrocatalyst [164]. In this work, a facile fabrication technology was carried out with graphitic carbon nitride (g-C_3_N_4_) and graphene oxide (GO) as raw materials. Another research group utilized N-doped graphene as an electrocatalyst in ORR under alkaline medium and in anion exchange membrane fuel cells [165].

## 3. Sulphur and Sulphur-Nitrogen Co-Doped Metal-Free Carbon Nanomaterials as Electrocatalysts

Nitrogen-doped carbon nanomaterials were accepted as potential electrocatalysts in ORR by researchers due to their charge transfer-induced performance using N contained in the graphitic framework, which further induced oxygen adsorption and reduction process at a comparatively lower overpotential value, which we briefly explained in the previous paragraph. In recent studies, other heteroatoms, viz., sulphur, phosphorus, boron and fluorine, were also studied to incorporate in carbon materials to promote their electrocatalytic activity in ORR as metal-free electrocatalysts in comparison to the undoped carbon nanomaterials [166,167,168,169]. However, dual doping of heteroatoms was believed to produce more effective electrocatalysts due to the synergistic effect between heteroatoms during ORR procedure in electrochemical performance and also in theoretical calculations. Their excellent synergistic effects induced the formation of higher numbers of catalytic sites with a remarkable reactant transport effect due to their hierarchical pore structures and greater electron transfer rate, which is generated by their three-dimensional continuous networked structures [170]. Li et al. mentioned that an optimal doping level could be the pivotal factor to control doping density and maximum catalytic performance in resultant materials [171]. In these types of nanomaterials, the total difference in electronegativity (d) generated from nitrogen and sulphur in comparison to what carbon might have generated indicates a more robust contribution of innovative nonelectroneutral sites in comparison to the monoheteroatom-doped structure (d_C_ = 2.55, d_N_ = 3.04 and d_S_ = 2.58). These special types of materials were reported to be more favourable to positively charged sites in the oxygen surface adsorption process, resulting in better ORR activity [172]. However, the reported works were mostly on doping of heteroatoms on CNTs and/or graphene, which could not be as effective for their high cost and complicated synthesis methodologies.

**Figure 11:**
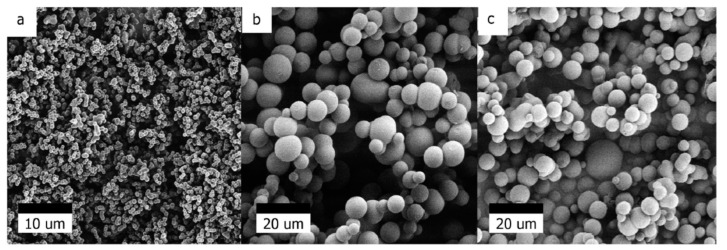
Scanning Electron Microscope images of products after hydrothermal carbonization method (**a**) pure glucose, (**b**) glucose with cysteine (Cys0.2) and (**c**) glucose with thienyl–cysteine (TCys0.2) (Reproduced with permission from Wohlgemuth et al. [174]).

In 2012, Wohlgemuth et al. reported the one-pot hydrothermal synthesis of sulphur-and-nitrogen-doped carbon aerogels which were utilized as potential electrocatalysts ORR [173,174]. In this method, two co-monomers, viz., S-(2-thienyl)-l-cysteine (TC) and 2-thienyl carboxaldehyde (TCA), were used in S incorporation using the typical solvothermal method. Further, the samples were placed inside a furnace under N_2_ atmosphere and were flushed for half an hour before being heated to 900 °C at a heating rate of 10 K min^−1^. This secondary pyrolysis step was applied in tuning carbon aerogel conductivity and heteroatom binding states. They had also conducted comparative electrocatalytic studies of these materials with solely N-doped aerogels in ORR both in acidic and alkaline mediums. They found co-doped materials to be better potential candidates in electrocatalytic study, which might be affirmed due to the synergistic effect between nitrogen and sulphur [173]. Figure 11 represented the micrographs of sulphur-and-nitrogen-doped carbon aerogels. 

Initially in 2002, the incorporation of sulphur into electrocatalytic performance was introduced by Wu et al. They had reported the development of sulphur-doped amorphous carbon as a potential cathode material by heat treatment of a mixture of polyacrylonitrile (PAN) and sulfuric acid [175]. Sulphur incorporation in the system enhanced the charge capacity value in close correlation with the increase in the size of graphite crystallites, interlayer distance and number of micropores. Choi et al. developed sulphur-doped carbons by using pyrolysis method of bioderived amino acids, viz., alanine, cysteine, glycine, niacin and valine, and utilized the materials as ORR electrocatalysts successfully in fuel cell applications. They had also synthesized a nitrogen-and-sulphur-co-doped catalyst by using cysteine, which performed best in acidic media in comparison to the commercially available Pt/C catalysts [176]. Previously, in 2006, Inamdar et al. introduced a new flame technology to synthesize spherical iron oxide nanoparticles by burning ferrocene solution using a spirit lamp [177]. The same research group synthesized carbon soot with various configurations using the flame pyrolysis method [178]. Thiophene was selected as the sulphur precursor material, and these nanomaterials were utilized as ORR electrocatalysts successfully. Park et al. reported high-quality S-graphene by using a lower content of oxygen-containing sulphur groups, with fluorinated graphite intercalation compounds (FGIC)-derived graphene as the starting material. This synthesis process was conducted at a comparatively lower temperature of 850 °C [179]. Zhang et al. developed graphene-doped N–S using cysteine as a nitrogen/sulphur source material that was potentially utilized in ORR with better performance than Pt/C. [180]. Similar work was reported by Zhao et al. to synthesize N/S-co-doped hollow carbon microspheres with great electrocatalytic performance in alkaline media [181]. Wang et al. used residues from banana peel to develop porous carbons, which were co-doped with N/S [182]. These materials were used as ORR electrocatalysts tested in alkaline medium. As we know, carbon can be derived from various sources by following simple methods. Cassava (Manihot esculenta) is a crop which can generate a large amount of waste material as peel and pulp. It contributes over 700 MT waste materials in global upstream food waste [183]. Duran et al. used cassava residues in preparation of sulphur-doped metal-free electrocatalysts through a thermal functionalization with sulfuric acid which were used in ORR under alkaline media [184].

## 4. Phosphorus- and Phosphorus–Nitrogen-Co-Doped Metal-Free Carbon Nanomaterials as Electrocatalysts

**Figure 12:**
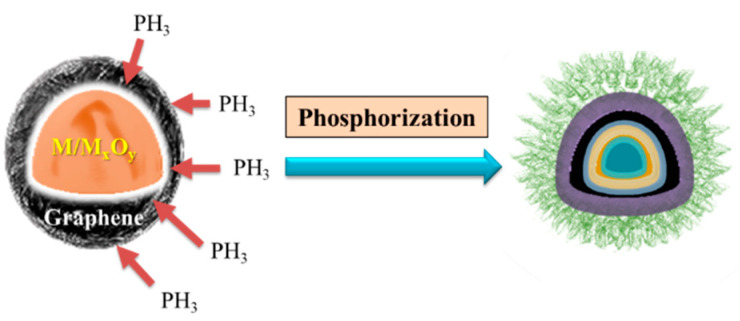
Schematic diagram of synthesis of P–doped, graphene-based, multilayered metal nanospheres by the solvothermal method.

**Figure 13:**
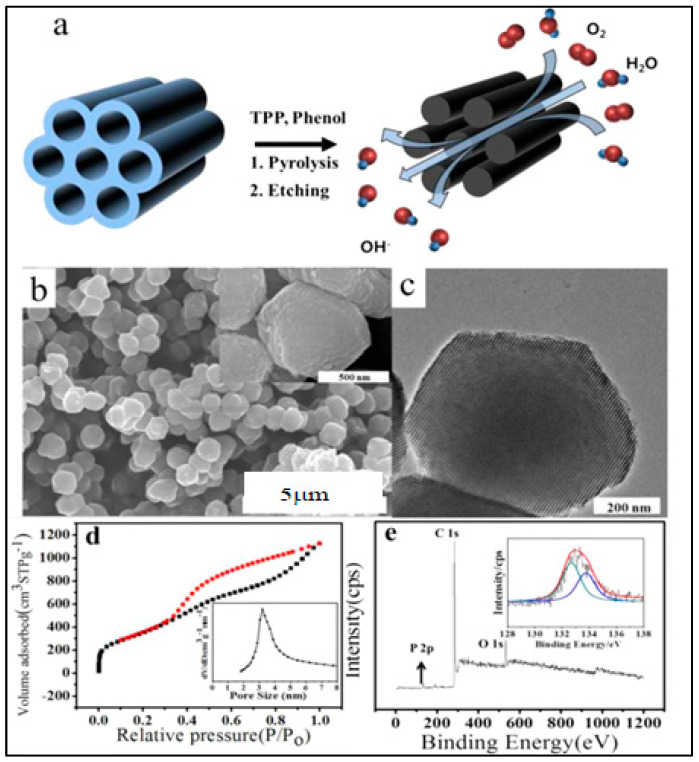
(**a**) Schematic illustration of POMC preparation, (**b**) FESEM image with an inset showing magnified image of POMC-3, (**c**) TEM image, (**d**) N2 sorption isotherms with an inset showing pore size distribution and (**e**) XPS survey scan and P 2p spectrum of POMC-3. (Reproduced with permission from Yang et al. [185]).

Phosphorus contains the same number of valence electrons as the nitrogen atom; therefore, it shows similar chemical properties [185], although it has a greater atomic radius value and higher electro-donating ability than nitrogen, which makes it a better choice as a dopant to carbon materials. Despite of these advantages, fewer research groups have been involved in the development of phosphorus-incorporated novel carbon nanomaterials with improved surface area and catalytic activity. Mostly, the reported studies were on phosphorus incorporation in metal–metal oxide substrate. Figure 12 represents a schematic diagram of P-doped, multilayered, graphene-based metal nanospheres using the solvothermal method. In 2012, Yang et al. demonstrated the novel structured, P-doped, ordered mesoporous carbon (POMC) as a potential electrocatalyst in alkaline medium for ORR [185]. In this typical process, a mixture solution of triphenylphosphine (TPP) and phenol was infiltered into an SBA-15 template at room temperature and pyrolyzed at 900 °C in Ar media, following the removal of the template by etching with HF to obtain silica-free POMC. The synthetic strategy with their micrograms and electrochemical studies is shown in Figure 13. In 2013, Prasad et al. reported the microwave-assisted one-pot synthesis of metal-free N-and-P-dual-doped nanocarbon as an electrocatalyst in ORR under alkaline medium [186].

**Figure 14:**
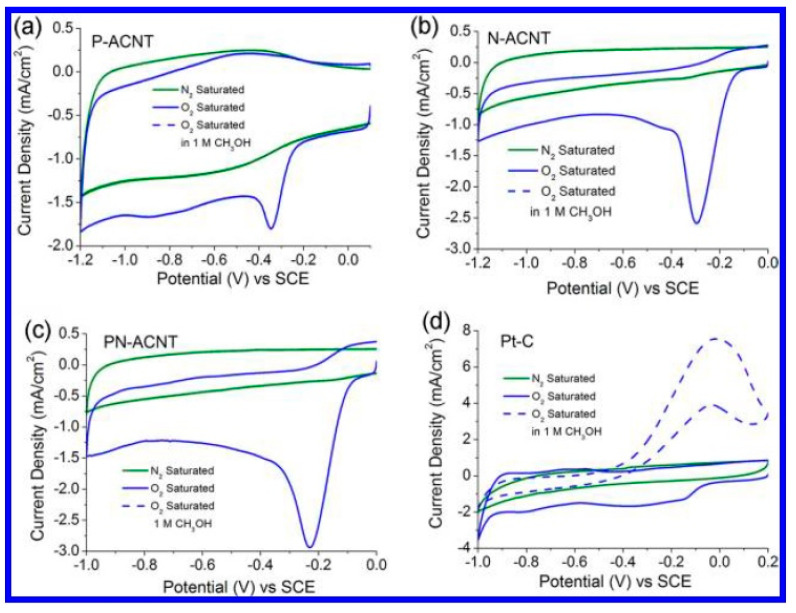
Cyclic Voltammograms for ORR in the presence of (**a**) phosphorus–, (**b**) nitrogen– and (**c**) phosphorus–nitrogen–doped CNTs; (**d**) Pt–Carbon electrocatalysts (Reproduced with permission from Yu et al. [191]).

In 2013, Li et al. synthesized P-doped graphene nanosheets by thermal annealing a homogeneous mixture of graphene oxide (GO) and BmimPF6 ionic liquid [187]. This innovative one-step method was economical and simple but scalable for mass production of P-doped graphene. These materials with phosphorous doping of 1.16 at% were proved to be better electrocatalysts in comparison to the commercial Pt/C catalyst in ORR under alkaline medium [187]. A similar method was adopted by Qiao et al. to develop N-and-P-dual-doped, reduced GO by pyrolyzing a homogeneous mixture of GO and diammonium hydrogen phosphate [188]. This material performed well as ORR electrocatalysts in alkaline medium following a four-electron pathway. A novel nanocasting method using platelet-ordered mesoporous silica as a template was adopted by Yang et al. in 2014 to develop P-doped, platelet-ordered, mesoporous carbon as excellent ORR electrocatalysts in alkaline media [189]. Nitrogen-and-phosphorus-co-doped, dual-doped hierarchical porous carbon foams were reported as efficient electrocatalysts in ORR developed through co-pyrolysis nitrogen- and phosphorus-containing precursors and poly(vinyl alcohol)/polystyrene (PVA/PS) hydrogel composites as in situ templates [190]. Yu et al. developed vertically aligned CNT arrays with co-doping of nitrogen and phosphorus using the chemical vapour deposition method [191], which exhibited excellent electrocatalytic stability and activity in ORR [191]. The electrochemical results had clearly shown enhanced electrocatalytic activity with co-doping (Figure 14).

## 5. Boron- and Boron–Nitrogen-Doped Metal-Free Carbon Nanomaterials as Electrocatalysts

Nitrogen- and phosphorus-doped carbon nanomaterials have been most extensively investigated in the literature, whereas S, Se, B and I have also been reported as potential electrocatalysts [192,193]. In 2011, Yang et al. reported synthesis of boron-doped carbon nanotubes as efficient electrocatalysts in ORR [194]. Many research groups reported the synthesis of boron-and-nitrogen-co-doped carbon nanomaterials, although their intrinsic synergistic effects have not been discussed yet. Zou et al. mentioned the influence of co-doping in catalytic applications through mathematical modelling [195]. However, a competent synthetic strategy should be established to anchor nitrogen and boron onto the appropriate sites of the carbon structure instead of establishing the N–B bond. Many researchers reported N,B-co-doped electrocatalysts, although their catalytic activity was not strong [196,197,198,199]. In contrast, high-performing electrocatalysts followed complicated synthetic strategies. Later, Zhou et al. synthesized a N,B-co-doped carbon catalyst with excellent electrocatalytic performance during ORR in alkaline media, in which nitrogen and boron were embedded into the carbon structure without establishing any bond between B and N [200].

## 6. Heteroatom Doped Activated Carbon as Electrocatalysts

Activated carbon (AC) has widely been employed as a main candidate for an electrode material in electrical double-layer capacitance (EDLCs) due to its high porous structure having greater surface area values (1000–3000 m^2^ g^−1^) and relatively greater packing density value of ~0.5 g cm^−3^ [201]. In the literature, many research groups synthesized activate carbon materials from bamboo, coconut shell, waste sawdust, etc. [202,203,204,205]. The hydrophobic characteristic of AC materials prevented the infiltration of the electrolytes in electrode materials, which further controlled the ion accessible surface area [206]. Moreover, the conductivity value of sp^3^ type AC is lesser than that of sp^2^ carbon, which resulted in lower capacitance [207]. The activated carbon was preferred over CNTs, MOFs, carbides and graphene, as later materials follow complicated, time-consuming and costly methodologies that restrict their large-scale application, regardless of containing higher surface area value with controllable pore structure and having high chemical stability [208]. On the other hand, graphene has very low volumetric capacitance value of less than 40 F/cm^3^, in comparison to that of 60 F/cm^3^ from activated carbon [209,210,211,212]. Chemical doping was an effective way to change the electronic and surface chemical properties of ACs [213]. The incorporation of single, dual or multiple heteroatoms into the carbon structure could enhance its physical and chemical properties to a significant extent [214]. In 2017, Fu et al. reported a highly efficient, low-cost approach to synthesize N-doped porous carbon materials from natural coconut silk fibres via a facile heat treatment and chemical activation. The resulting activated carbon showed excellent catalytic activity and admirable stability for the ORR in alkaline media [215]. Another research group developed the high-performing P-doped activated carbon as a catalyst in air-cathode microbial fuel cells [216]. In this case, phosphorus was present as C–O–P groups, which creates a greater power density value and better electrocatalytic performances [216]. It was reported that AC had shown poor specific capacitance due to having an extensive range of pore size distribution that contradicted with the supercapacitor requirement that pore size should match to the size of electrolyte ions. The hydrophobic nature of ACs resulted in a limitation of ion-accessible surface area due to poor infiltration of electrolyte-to-electrode materials [217]. The optimization was required between pore size, pore structure, surface properties and electrodes’ conductivity to enhance maximum capacitance [218]. In this regard, the role of incorporation of nitrogen into ACs was mentioned by Zheng et al. They found a linear relationship between the specific surface area and nitrogen content in functionalized AC towards the capacitance value [219]. Incorporation of heteroatoms in ACs could also be explained through defect mechanism, which was to design defective carbons for ORR [220]. This concept implied that if the desired defect could be produced in AC structure, it could become an active electrocatalyst in ORR. In this regard, Yan et al. employed a simple N-doping and removal method, making the inert activated carbon highly active for both the ORR and HER by creating unique defects in the AC [221].

## 7. Conclusions

The heteroatom-doped nanomaterials have drawn huge attention in last two decades in the field of nanoscience and nanotechnology due to their synthetic methodologies, unique properties and potential electrochemical performances through the synergistic effects between the heteroatoms with carbon. The introduction of defects into the carbon-based materials could easily change the distribution of electron density and electronic charge, which could result in remarkable progress in the catalytic performance in various electrochemical reactions. In comparison to the undoped carbon nanomaterials, these specially disordered doped and co-doped materials performed remarkably well as electrocatalysts due to their larger functional surface area value and greater ratio of surface-active groups to volume. This review article covered the most popular potential synthetic methodologies to develop nitrogen, sulphur, phosphorus, boron and their multiple doped, metal-free carbon nanomaterials and discussed elaborately about their electrocatalytic activity in various electrochemical process. The results have clearly shown the enhancement of nanomaterials’ electrocatalytic activity due to co-doping of multiple heteroatoms into carbon nanostructures, affirmed from the synergistic effect between them. Despite the abundant studies on this topic, many scientific questions are yet to be solved. No such doping methodology has yet been developed that could precisely control the locations of the dopant and its domain sizes. Moreover, it is not clearly understood how the supporting environment interacts with catalytic active centres in these materials. Despite of the challenges, these specially structured nanomaterials exist with many avenues and require more progress in carbon-based nanotechnology with applications in the energy field.

## Figures and Tables

**Table 1:** Heteroatom-incorporated metal-free carbon nanomaterials, synthesis and application in last two decades.

Year	Materials	Synthesis Method	Application
2009	N-doped carbon nanotubes	Chemical vapor deposition method	ORR in fuel cell application [50]
2010	N-doped graphene	Chemical vapor deposition method	ORR in fuel cell application [51]
2011	N-doped carbon nanotubes	Chemical vapor deposition method [52], Amine Flames [53], Chemical vapor deposition method [54]	ORR in microbial fuel cells [52], Supercapacitors [53]
2012	(i) N-doped graphene foam, (ii) N-doped graphene quantum dots	(i) Postsynthesis annealing in ammonia (ii) Solution Chemistry	(i) Dye-sensitized solar cells [55], (ii) ORR [56]
2013	N-doped carbon nanomaterials	Solvothermal process	Water oxidation [57]
2014	C_3_N_4_@NG	Chemical vapor deposition method	HER electrocatalysts [58]
2015	(i) Nitrogen-doped graphene/CNT composite, (ii) N,P-doped carbon foam, (iii) Carbon nanocages	(i) Modified Hummers’ method for the GO fabrication [73] (ii) Pyrolysis of a polyaniline aerogel (iii) Hard templating method	(i) ORR in acidic fuel cell [59], (ii) ORR and OER [60], (iii) ORR [61]
2016	N,P-co-doped carbon networks	Soft template and Pyrolysis	ORR and HER [62]
2017	Carbon-based metal-free nanomaterials	Solvothermal process	OER, ORR and HER [63]
2018	(i) N-doped hierarchical porous carbon nanosheets, (ii) N-doped porous carbon, (iii) MOF-derived nitrogen-doped highly disordered carbon	(i) Template-free method, (ii) Pyrolysis, (iii) Solvothermal method	(i) ORR [64], (ii) Electrocatalytic N_2_ reduction [65], (iii) Electrochemical synthesis of ammonia (ESA) through the nitrogen reduction reaction (NRR) [66]
2019	Nitrogen-doped carbon-based catalysts	Solvothermal method	Acidic oxygen reduction [67]
2020	(i) Tellurium-doped, mesoporous carbon nanomaterials, (ii) Nitrogen-doped metal-free nanomaterials, (iii) Nitrogen-doped metal-free nanomaterials	(i) Pyrolysis, (ii) Solvothermal, (iii) Solvothermal	(i) Bifacial dye-sensitized solar cells [68] (ii) Bifunctional oxygen electrocatalyst for ultrastable zinc-air batteries [69], (iii) Selective catalytic oxidation of hydrogen sulphide [70]
2021	(i) Oxygen- and nitrogen-doped metal-free microalgae carbon nanoparticles, (ii) Nitrogen-doped graphene/CNT composite	(i) Potassium hydroxide (KOH) activation of Spirulina Platensis microalgae, (ii) Pyrolysis	(i) Hydrogen production from sodium borohydride in methanol [71], (ii) ORR in acidic fuel cell [72]

## Data Availability

Not Applicable.
